# A comparative study of stereo-dependent SSVEP targets and their impact on VR-BCI performance

**DOI:** 10.3389/fnins.2024.1367932

**Published:** 2024-04-09

**Authors:** Haifeng Liu, Zhengyu Wang, Ruxue Li, Xi Zhao, Tianheng Xu, Ting Zhou, Honglin Hu

**Affiliations:** ^1^School of Information Science and Technology, ShanghaiTech University, Shanghai, China; ^2^Shanghai Advanced Research Institute, Chinese Academy of Sciences, Shanghai, China; ^3^School of Microelectronics, Shanghai University, Shanghai, China; ^4^Shanghai Frontier Innovation Research Institute, Shanghai, China; ^5^University of Chinese Academy of Sciences, Beijing, China

**Keywords:** virtual reality (VR), brain-computer interface (BCI), steady-state visual evoked potentials (SSVEP), three-dimensional (3D) stimuli, binocular vision

## Abstract

Steady-state visual evoked potential brain-computer interfaces (SSVEP-BCI) have attracted significant attention due to their ease of deployment and high performance in terms of information transfer rate (ITR) and accuracy, making them a promising candidate for integration with consumer electronics devices. However, as SSVEP characteristics are directly associated with visual stimulus attributes, the influence of stereoscopic vision on SSVEP as a critical visual attribute has yet to be fully explored. Meanwhile, the promising combination of virtual reality (VR) devices and BCI applications is hampered by the significant disparity between VR environments and traditional 2D displays. This is not only due to the fact that screen-based SSVEP generally operates under static, stable conditions with simple and unvaried visual stimuli but also because conventional luminance-modulated stimuli can quickly induce visual fatigue. This study attempts to address these research gaps by designing SSVEP paradigms with stereo-related attributes and conducting a comparative analysis with the traditional 2D planar paradigm under the same VR environment. This study proposed two new paradigms: the 3D paradigm and the 3D-Blink paradigm. The 3D paradigm induces SSVEP by modulating the luminance of spherical targets, while the 3D-Blink paradigm employs modulation of the spheres' opacity instead. The results of offline 4-object selection experiments showed that the accuracy of 3D and 2D paradigm was 85.67 and 86.17% with canonical correlation analysis (CCA) and 86.17 and 91.73% with filter bank canonical correlation analysis (FBCCA), which is consistent with the reduction in the signal-to-noise ratio (SNR) of SSVEP harmonics for the 3D paradigm observed in the frequency-domain analysis. The 3D-Blink paradigm achieved 75.00% of detection accuracy and 27.02 *bits*/*min* of ITR with 0.8 seconds of stimulus time and task-related component analysis (TRCA) algorithm, demonstrating its effectiveness. These findings demonstrate that the 3D and 3D-Blink paradigms supported by VR can achieve improved user comfort and satisfactory performance, while further algorithmic optimization and feature analysis are required for the stereo-related paradigms. In conclusion, this study contributes to a deeper understanding of the impact of binocular stereoscopic vision mechanisms on SSVEP paradigms and promotes the application of SSVEP-BCI in diverse VR environments.

## 1 Introduction

Brain-computer interface (BCI) (Wolpaw et al., 2000) is an emerging interdisciplinary technology that establishes new information pathways between the brain and the external environment (Yu et al., 2019), which can be achieved without relying on peripheral nerves or muscles, and realizes direct interaction between the brain and external devices. Electroencephalography (EEG)-based BCIs (Lotte et al., 2018) have gained significant attention in recent years due to their ease of deployment and high temporal resolution, typical paradigms of which are P300 (Xiao et al., 2021), Steady-State Visually Evoked Potentials (SSVEP) (Yin et al., 2015), and Motor Imagery (MI) (Jin et al., 2020). Among these paradigms, SSVEP-BCI stands out due to its advantages of high Information Transfer Rate (ITR) (Cheng et al., 2002) and Signal-to-Noise Ratio (SNR) (Wang et al., 2016), as well as its low requirements for subjects, which collectively make it one of the easiest BCI paradigms to deploy and apply at present.

Human EEG signals are inherently variable and subject to noise. However, a specific frequency of luminance-based flickering stimulation can elicit EEG signals with distinct frequency characteristics. This phenomenon is known as SSVEP (Kaspar et al., 2010). SSVEP is usually characterized by a stable and synchronized response in the brain at the stimulation frequency. In a typical SSVEP-BCI system, a liquid crystal display (LCD)/light-emitting diode (LED) computer screen is used to simultaneously present several stimuli flashing at specific frequencies (Mu et al., 2020). EEG signals are collected during the presentation of these stimuli and then processed and classified as instructions for BCI control (Allison et al., 2012).

In early SSVEP experiments, LEDs were commonly used to directly present physical visual stimuli. However, the mature and mainstream approach in the current time mainly involves the use of CRT/LCD displays to present various planar stimuli (Wang et al., 2016; Liu et al., 2020). Since there has been research on SSVEP using an LCD display to present 3D stimuli in the past (Mun et al., 2013), there are emerging studies in this field that have begun exploring similar integrative applications using Virtual Reality (VR)/Augmented Reality (AR) devices (Chen et al., 2021; Mahmood et al., 2022; Zhang et al., 2023). Unlike traditional LCD displays, VR/AR is a binocular vision-based display device that uses parallax and immersive computer graphics effects to build an interactive environment. Despite extensive research (Anzai et al., 1997; Kim et al., 2016) on stereoscopic and depth perception in neurology, few studies have been conducted on BCI systems that use stereo-related visual stimuli. Han et al. (2019) proposed a novel stimulation method for VEP-BCI based on stereoscopic motion, which utilizes the mechanism of binocular parallax.

However, there are emerging paradigms in the field of SSVEP, such as illusion-induced visual evoking potential (IVEP) (Li et al., 2023) and smoothed steady-state motion visual evoking potential (SSMVEP) (Yan et al., 2017), which incorporate human motion perception into SSVEP-based BCI systems, offering brand new directions for SSVEP-BCI technology. The results of the SSMVEP experiments demonstrate that the CCA features of stereoscopic EEG signals are significantly stronger compared to nonstereoscopic motion, and more regions of the brain were activated (Guo et al., 2022). Likewise, the exploration of visual stimulation combined with stereo perception in brain-computer interfaces holds significant potential for advancing the field and merits further investigation.

Currently, there have been limited studies that directly explore the combination of stereoscopic stimuli and the SSVEP-BCI paradigm. However, some studies have started investigating the effects of stereoscopic scenes and stereoscopic visual stimuli on BCI systems. Qu et al. (2018) proposed a novel P300 EEG speller that utilizes stereo visual stimuli, which generated higher amplitude P300 waveforms compared to the traditional 2D P300 speller. The results of the ERP analysis of this study revealed the impact of stereoscopic stimulation on EEG. However, in their study, complete stereoscopic stimuli were not utilized and specific experiments targeting stereoscopic perception were not designed. Zehra et al. (2023) compared AR-based SSVEP with 3D and 2D stimulation using three different strategies: flickering, grow-shrink, and both. However, the use of dry electrodes and the characteristics of AR perspective led to interference by various factors, resulting in overall low performance. Zhu et al. (2023) examined the effects of shape, color, and frequency parameters on 3D SSVEP in a VR setting, but without specifically focusing on comparing the characteristics or performance differences between 3D stimulation and flat stimuli.

In this study, we mainly investigate the impact of stereoscopic perception on the performance of SSVEP-BCI under a virtual reality environment through a comparative analysis with 3D stereoscopic stimuli and 2D plane stimuli presented under identical conditions. Attempts were also made to construct new paradigms to combine stereoscopic perception and visual evoked potentials to make SSVEP-BCI more suitable for 3D space applications. A new VEP approach is proposed, which involves the periodic disappearance and reappearance of a stereoscopic object at a fixed position through inversion of opacity instead of luminance. Comparing to existing studies, this study examines the distinctions between traditional 2D stimulation and stereo-related stimulation in VR environments by analyzing frequency domain features and evaluating the performance of training and non-training recognition methods. The findings offer insights for advancing spatial interaction paradigms.

The remaining paper is arranged as follows: Section 2 introduces the materials and methods, which mainly describe the setup of the VR-BCI system and the experimental design. Section 3 reports the experimental results, and Section 4 discusses the significance and future directions of this work. Finally, the conclusion is presented in the last section.

## 2 Materials and methods

### 2.1 VR-BCI system setup

**Figure 1:**
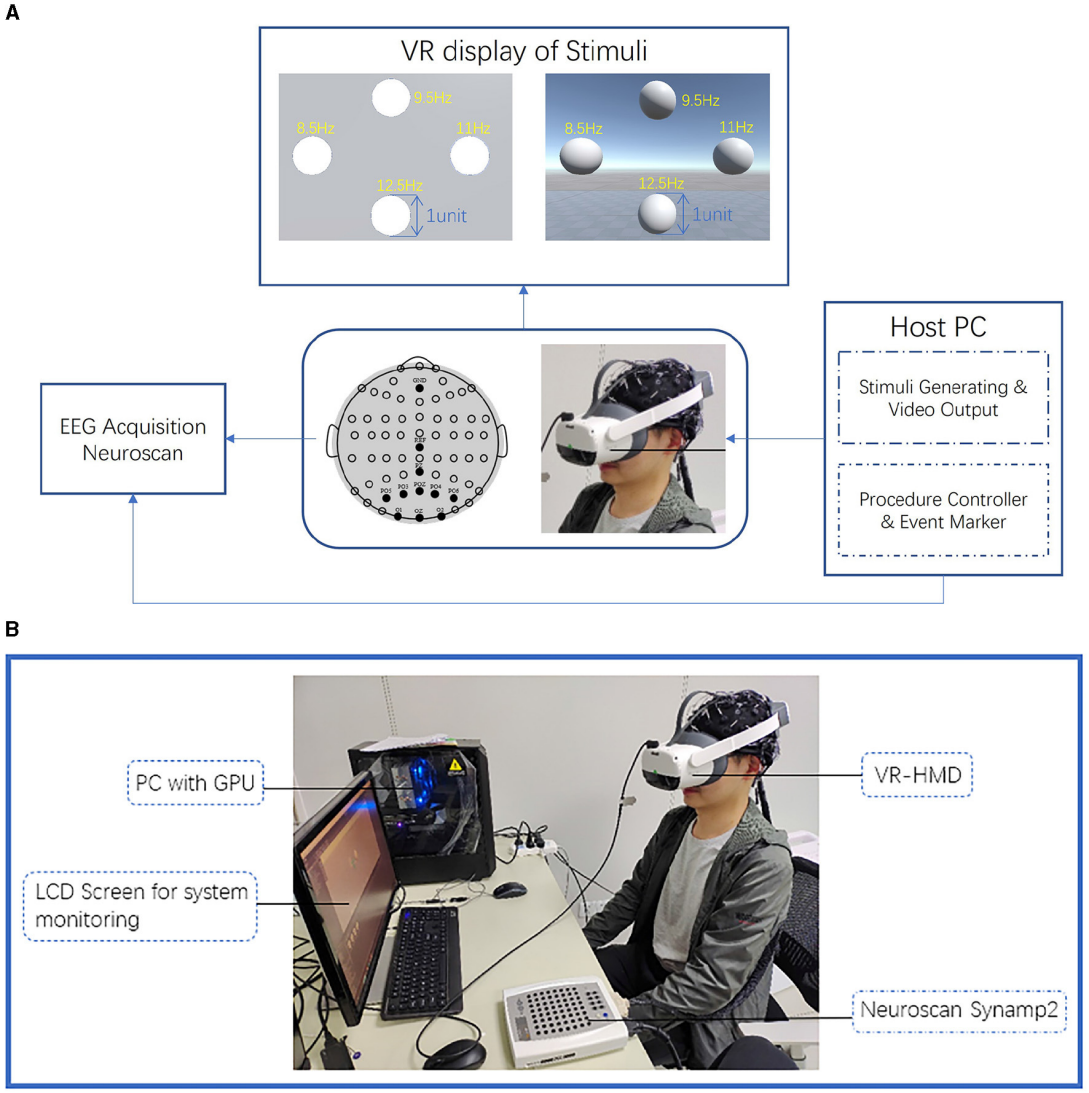
Experiment setup. **(A)** Schematic diagram of the experiment system. The size, position, and parameters of all four objects in different experiment scenes are exactly the same, with a length unit of 1m in the default unity scale. **(B)** VR-BCI system setup. Visuals are transmitted to the VR headset through a direct display port connection using a desktop computer equipped with a GPU, which simultaneously controls the event markers of Neuroscan.

In this study, we developed a combined VR-BCI system to present both stereoscopic and planar stimuli, collecting the EEG signal mainly from the parietal and occipital lobes. The framework of this system, as shown in Figure 1, can be mainly divided into three parts: stimulus presentation, EEG acquisition, and EEG signal processing. The central control unit of the system is a PC with a GPU, running the Unity3D program (Unity Technologies, San Francisco, CA, USA), connecting VR-HMD (PICO Neo3 Pro, PICO Interactive Inc, Shanghai, China) through a DP cable connection and presenting stimuli with steamVR (Valve Corporation, Bellevue, WA, USA). A Neuroscan SynAmps2 system was set up for continuous EEG acquisition, equipped with a 64 channel electrode cap according to the international 10-20 system at a sampling frequency of 1, 000 Hz. The host PC is connected to the Neuroscan device through a parallel port, allowing the Unity experimental program to provide accurate markers directly based on event triggers.

The internal display of the PICO Neo3 Pro has a resolution of 2,160 × 1,200 (1,080 × 1,200 per eye) pixels and a vertical refresh rate of 90 Hz. The PICO Neo3 Pro offers a 98-degree field of view and optical adaptation for the adjustment of the interpupillary distance (IPD) in the range of 55 − 71 mm, providing participants with a fundamentally satisfying 3D visual experience.

To ensure precise timing synchronization for the experiment, PICO Neo3 Pro is configured to use a Display Port (DP) connection mode to directly receive video output from a desktop computer with a dedicated graphics card (GeForce RTX3060, NVIDIA), allowing seamless transmission of visual content. The physical update interval of Unity Engine is adjusted to 1 ms, which is required for high-precision synchronization in certain scenarios. In the described system, using a fixed duration of stimuli presented at regular intervals and sending event markers through the parallel port can indeed minimize the impact of network latency on the overall system.

### 2.2 EEG acquisition

#### 2.2.1 Participants

Ten subjects (nine male and one female, aged 21–26) from ShanghaiTech University and Shanghai Advanced Research Institute (Chinese Academy of Sciences) were recruited to participate in the experiments in this study. All subjects had normal or corrected to normal binocular vision, with the ability to perceive stereoscopic perception, and without reported issues of neurological disease or impairments. Participants had not received specific training for the experiment prior to this experiment, although some of them had previous experience participating in similar SSVEP experiments. All subjects received informed consent under the method and procedure approved by the Ethics Committee of ShanghaiTech University. Subjects were required to follow the corresponding experimental instructions and remain attentive during the experiment. Data from ten subjects were included in the final analysis.

#### 2.2.2 EEG recordings

Experiments were implemented in an electromagnetically shielded room to eliminate external noise. Nine electrodes overlaying parietal and occipital areas (*Oz, O*1, *O*2, *Pz, POz, PO*3, *PO*4, *PO*5 and *PO*6) (Wang et al., 2016; Nakanishi et al., 2017) were used to collect continuous EEG signals elicited by visual stimuli generated by VR-HMD, with the Neuroscan SynAmps2 system. We placed the reference electrode in the central area and the ground electrode in the frontal area. All electrode impedances were kept below 10*kΩ*. In order to facilitate the extraction of relevant event data and enable subsequent analysis, the presentation of stimuli was synchronized with event markers on the EEG data, which were simultaneously transmitted from the desktop computer to the SynAmps2 system with parallel port.

### 2.3 Stimuli and experiment design

An experiment was designed to investigate the impact of stereo-related objects on the performance of SSVEP-BCI. The classification performance of planar SSVEP objects and stereoscopic SSVEP objects was directly compared in a typical 4-target selection task, while different implementations for visual stimuli were employed.

For each subject, all tasks were performed on the same day but during separate sessions. Between sessions, we provided participants with ~5–10 min of rest to readjust to a natural state without wearing the VR headset, which aimed to exclude possible influences like 3D dizziness. A regular break of 30 s was also provided between consecutive blocks within each session. The experiment began with a briefing session where participants received an introduction to the study objectives and instructions on performing the tasks.

Due to the challenges in controlling the consistency of the stimulus area and the distance between the PC display and the VR display, all experiments in this study were carried out within VR scenes. The diameter of the designed round or spherical stimulus was adjusted to a default of 1 unit in the Unity engine. Additionally, the subjects' viewing angle distance was set at four units of stimulus distance within the scene. By utilizing VR technology, our goal was to ensure a standardized condition for experiments and eliminate potential variations that may arise from physical displays and their respective distances.

After each participant completes all the tasks, we administer a questionnaire to obtain feedback on the comfort level of different stimulus presentation methods, as well as the general evaluation of factors such as clarity of the visuals during the VR experiment, the presence of 3D motion sickness, comfort of wearing the equipment, and fatigue experienced during the duration of the experiment.

#### 2.3.1 Experiment design: 4-target selection task

**Figure 2:**
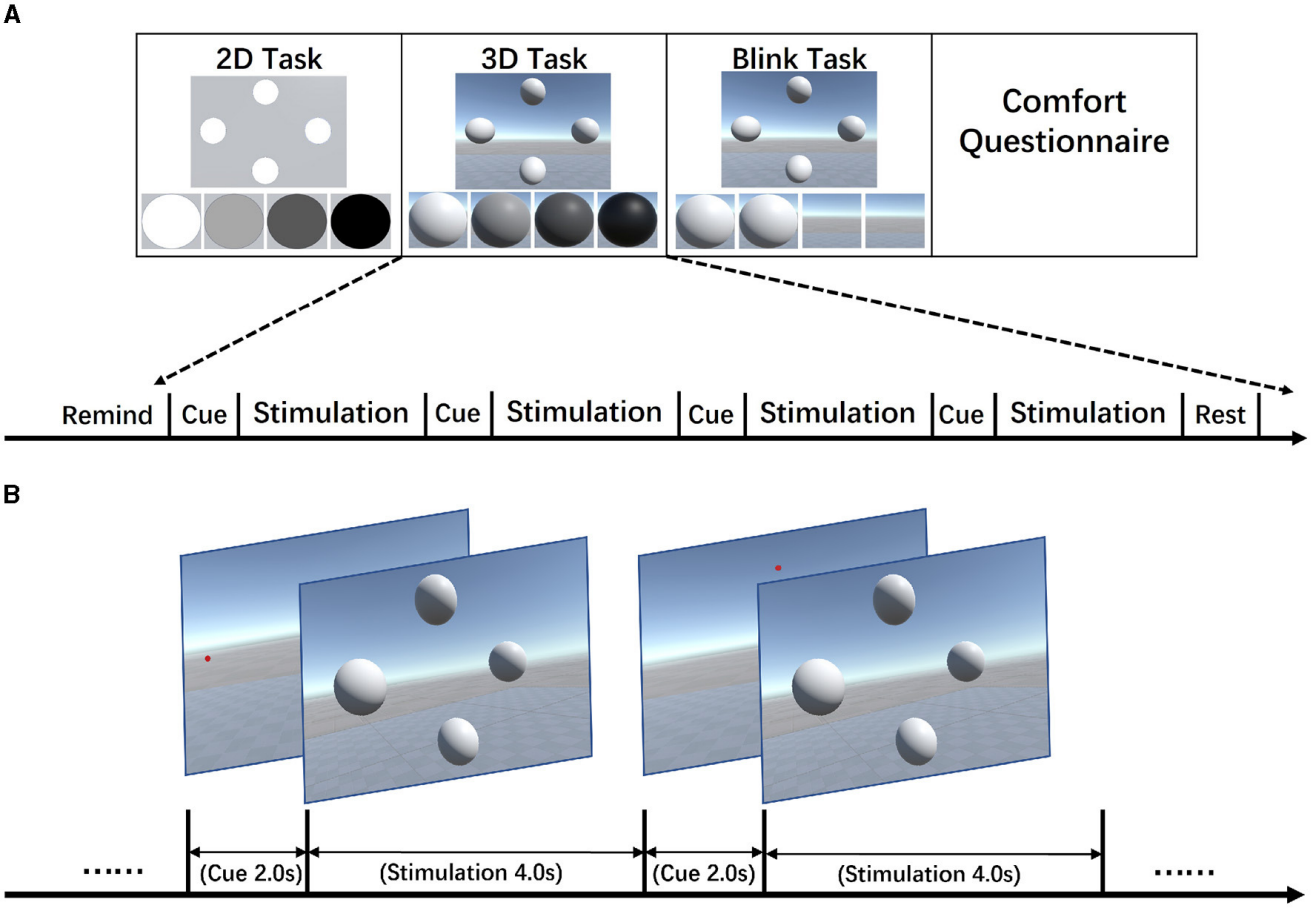
Experiment protocol. **(A)** Experiment tasks design. The four-target selection tasks were designed using identical methods for all types of stimulus presentation. The different tasks are performed in separate sessions, resulting in three sessions for each participant's experiment. Each session consists of three blocks, with each block comprising 20 trials, totaling 60 trials. Participants were given 10 s of preparation time at the beginning of a session. A 30-s rest period was provided after each block of multiple consecutive tests. After completion of all three tasks, a questionnaire was administered to obtain feedback from the participants. **(B)** Experiment procedure. In each trial, all four stimuli were presented simultaneously during the simulation period. One trial consists of a 2-s display of the target location followed by a 4-s stimulus presentation. Following each trial, the next target would be switched, and the switching order starts from the target at 8.5 Hz and proceeds clockwise through the four targets, which would finally complete one round of the 4-target recognition task. The entire experiment consists of repeating this process multiple times.

As shown in Figure 2, based on the standard SSVEP four-target selection task, the experiment involved a baseline 2D task and two 3D stereoscopic stimulus task using the sinusoidal mode and the Blink mode, with each task consisting of 60 trials to investigate the differences between the three types of stimuli. Figure 2 illustrates the experimental content and provides an overview of the basic procedure. Each trial in the experiment consisted of a 2-s cue period followed by a 4-s simulation period.

**Figure 3:**
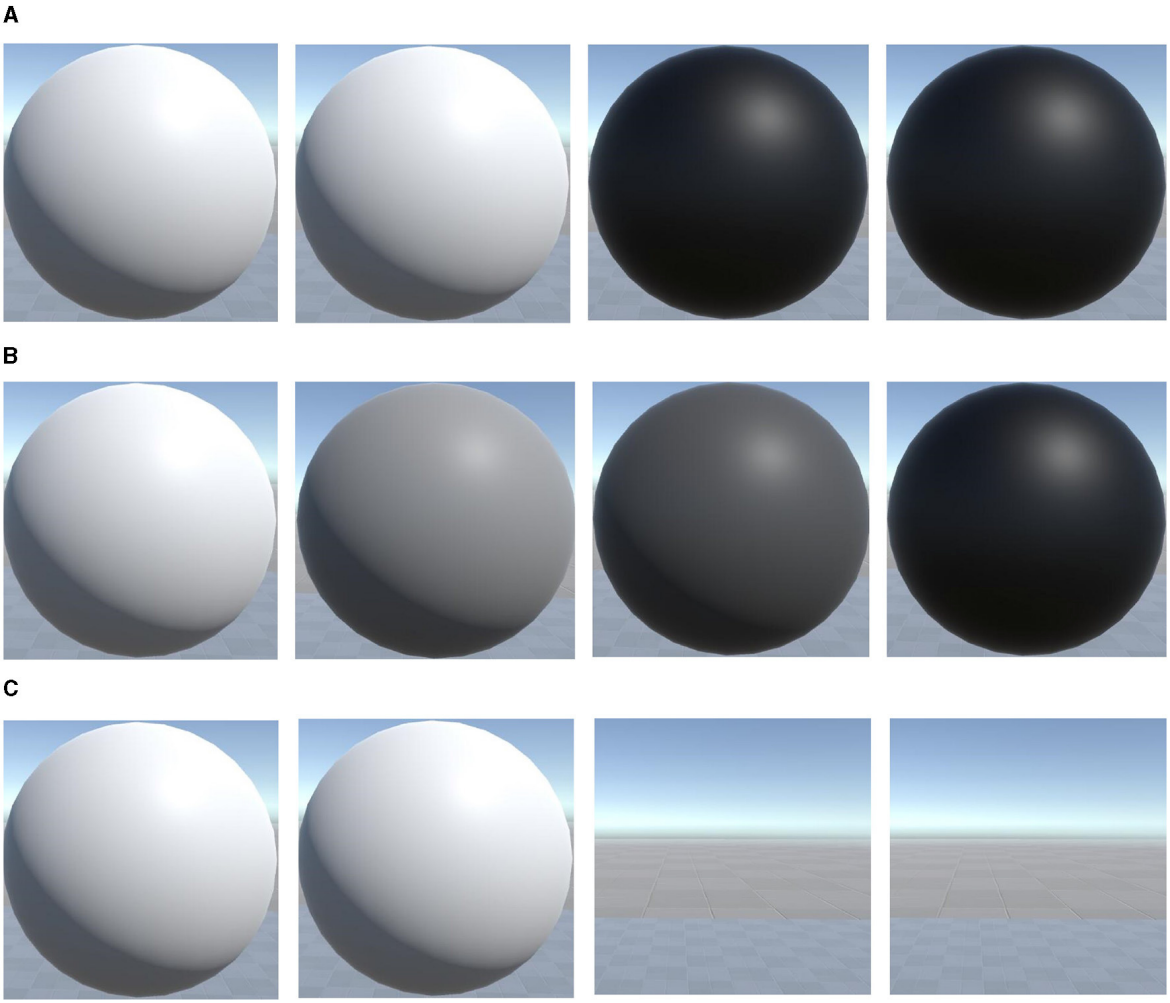
Three different implementations of visual stimuli. **(A)** Inversion of Luminance. **(B)** Sinusoidal Modulation of Luminance. **(C)** Inversion of Opacity.

There were a total of three sessions, and each session included three blocks. Within each block, the four target selections were conducted five times, which means one block consisted of 20 trials. During each cue period, a red indicator dot appeared at one of four specific locations, which were the center positions of the stimuli shown in Figure 2. Subjects were instructed to direct their gaze toward the position of the red dot whenever it appeared and to maintain a steady gaze during the stimulation period. All four targets are flickering simultaneously during each simulation period. In the VR scene design, four stimuli were arranged in a cross pattern on a vertical plane located at a distance of two standard units from the camera's viewpoint, as shown in Figure 2A. The distances between the upper and lower stimuli, as well as between the left and right stimuli, were set to three standard units. The stimuli themselves were circular planes or spheres with a diameter of 1 standard unit. During the actual experiment, the participants could comfortably maintain their gaze on each stimulus, even after making slight adjustments to their viewing angles. The 2D and 3D stimuli in the VR scene were rendered using the default Unity material, but 3D stimuli had lighting and shading effects additionally. Cotrina et al. (2017) discussed the impact of switching between different points of focus on the effects of SSVEP. As the primary focus of this research was not on the potential effects of focal switching, a gray plane positioned at a fixed distance in front of the observer was used to present the 2D stimuli. This ensured consistent effects with the PC-SSVEP paradigm while eliminating potential confounding factors. For 3D tasks, the positions of the 3D spheres' centers are also on the same plane as the center of the 2D circles. However, in 3D tasks, this plane is not displayed, and the background maintains a natural 3D skybox environment as shown in Figure 2A. In the overall experimental task, the 2D, 3D and 3D-Blink tasks were conducted in separate sessions. Within each session, the four stimuli flickered at fixed frequencies as 8.5, 9.5, 11, and 12.5 Hz, positioned as shown in Figure 1A, starting with the upper object and in clockwise order. We chose these frequencies based on Wang et al. (2016) and similar AR-SSVEP studies (Zhang et al., 2023), also with the consideration about fresh rate of VR device. We developed two methods for presenting flickering stimuli in the Unity3D program, as shown in Figures 3A, B. Flip mode reversed colors at fixed intervals, whereas sinusoidal mode used a modulated sine wave for smooth luminance transitions, with flip mode being more affected by hardware refresh rate and sinusoidal mode being more robust to dropped frames. In this study, to address the accuracy issues with the stimulus frequency and event markers in Unity, the update time of the engine was adjusted to 1*ms*. Additionally, the sinusoidal mode was used by default for presenting both 2D and 3D stimuli, as shown in Figure 3B, to minimize the impact of error factors such as dropped frames.

#### 2.3.2 SSVEP paradigms

In this experiment, we compared three different paradigms: 2D, 3D, and 3D-Blink, using the same target selection task. The 2D and 3D paradigms presented stimuli of the target frequency through sinusoidal modulation of luminance. We aimed to investigate the influence of planar and stereoscopic stimuli on the SSVEP features through comparison of their results. Due to the primary source of immersion and depth perception in VR being binocular display-induced disparity, we came up with a novel approach to present visual stimuli, as shown in Figure 3A, to further leverage this visual characteristic to construct visual stimuli that are better aligned with the SSVEP paradigm. This new simulation mode, which we named 3D-Blink, modified the original luminance-related modulation of SSVEP stimuli by using inversion of opacity instead of luminance to achieve a periodic appearance and disappearance of the target at a specific frequency, which overcame the discomfort caused by luminance flickering and might further benefit from depth perception.

### 2.4 EEG processing

All the original signals were downsampled to achieve a sampling rate of 250 SPS and filtered using a notch filter with a frequency range of 48 − 52 Hz to eliminate power frequency interference. The desired frequency band was obtained through the implementation of a second-order Chebyshev IIR filter with a passband ranging from 4 to 40 Hz.

### 2.5 Classification methods

#### 2.5.1 Filter bank canonical correlation analysis

As the most classic SSVEP method, Canonical Correlation Analysis (CCA) (Lin et al., 2006) analyzes SSVEP signals by calculating the canonical correlation coefficients of the two groups of signals. While CCA can generally reflect the effectiveness of the implementation of the SSVEP system, its performance is not sufficiently stable to meet real-time requirements for accurate interaction. Compared to ordinary CCA, FBCCA (Chen et al., 2015) can better leverage potential gains from harmonics of the target frequency. By incorporating a filter bank mechanism, FBCCA can decompose the input signal into multiple frequency bands and process each band independently, and then the CCA results of all frequency bands are weighted and superimposed to produce the final FBCCA results. This approach enables FBCCA to better utilize the SSVEP feature information contained in EEG signals, thereby improving the overall signal-to-noise ratio, which makes FBCCA one of the best non-calibration algorithms for SSVEP. The FBCCA method in this study processed 3 sub-bands, namely 6 − 90 Hz, 14 − 90 Hz, and 22 − 90 Hz, with the number of harmonics set to 3. The calculation method used for the weights of the sub-band components is *w*(*n*) = *n*^−*a*^+*b*, where *n*∈[1, *N*], *n* is the sub-band index, and *N* is the total number of sub-bands. The FBCCA algorithm employed in this study uses 1.25 and 0.25 as the parameter values for *a* and *b*, respectively, referring to previous studies (Chen et al., 2015).

#### 2.5.2 Task-related component analysis

Based on previous research, deep perception can elicit specific ERP (Event-Related Potentials) responses (Guo et al., 2022). In our study, we hypothesized that 3D stimuli could also evoke these ERP components in EEG data. Due to the time-locking nature of these ERP components, TRCA (Nakanishi et al., 2017) is a suitable classification method for extracting and utilizing potential ERP characteristics related to stereoscopic stimuli. TRCA constructs spatial filters to maximize task-related correlations in multiple time series. By utilizing the reproducibility of task-related components, TRCA significantly improves the signal-to-noise ratio of SSVEP. Unlike methods such as CCA, TRCA does not require high accuracy in the frequency of the stimuli. Instead, its focus lies on the consistency of the stimulus components in each task, emphasizing accurate time synchronization, especially for strongly time-locked EEG features. The TRCA method used in this study employed the same filter bank strategy as the FBCCA method.

### 2.6 Performance evaluation

#### 2.6.1 Amplitude and signal-to-noise ratio

Amplitude values reflect the intensity or magnitude of the SSVEP response. By comparing the amplitudes between 2D and 3D stimuli, we can assess if depth perception induced by the 3D stimuli leads to larger or different SSVEP responses compared to 2D stimuli. SNR is a crucial indicator for evaluating the quality of SSVEP signals and is widely used in SSVEP research (Wang et al., 2016; Zhang et al., 2022). As a measure of the quality of the SSVEP signal, SNRs indicate the strength of the signal relative to background noise. Higher SNR values suggest a more reliable and accurate detection of SSVEP responses. In this study, we defined the SNR as the ratio between the amplitude at frequency *f* Hz and the average amplitude within the frequency bands [*f*−2, *f*) Hz and (*f, f*+2] Hz (Zhang et al., 2022).

#### 2.6.2 Information transfer rate

Information transfer rate (Cheng et al., 2002) is widely used to evaluate communication performance in most previous BCI studies. In the offline experiment, we evaluated the accuracy of the target recognition algorithm by varying the data length from 0.2 to 2 s with a step size of 0.2 s. After comparing the recognition accuracy of different paradigms, we selected the one with the best performance and the best ITR. These evaluations allow us to determine the optimal data length and assess the overall effectiveness of the system. ITR can be computed with:


(1) $$\begin{array}{l} \text{ITR}=\left(\log_{2}\left(N\right)+p\log_{2}\left(p\right)+\left(1-p\right)\log_{2}\left(\frac{1-p}{N-1}\right)\right)\times \frac{60}{T}, \end{array}$$

where *N* stands for the number of targets, *p* is the target identification accuracy, and *T* is the time per selection (including the length of the time required for gaze shifting and time of the whole stimulus progress).

#### 2.6.3 Comfort evaluation

VR-SSVEP, compared to traditional PC-SSVEP, uses closer displays to create a sense of stereoscopic vision and immersion. Although the field of view angle is limited, the design of object placement in VR allows for more flexibility. In a previous experiment, participants found VR-SSVEP more comfortable and reported a softer perception of stimuli during the stare-to-switch process. To assess the comfort level and usability of the paradigms, we used experiment feedback questionnaires and rating scales. For some users, VR headsets can cause motion sickness and visual fatigue due to a mismatch in interpupillary distance (Mun et al., 2012). Therefore, to obtain a comprehensive assessment of the user experience in VR-SSVEP applications, we took into account the overall experience of the participants and conducted a survey to identify factors that influenced their perception.

## 3 Results and analysis

### 3.1 Feature analysis

The objective of this study is to investigate the differences in SSVEP signals induced by 2D and 3D stimuli in a virtual environment. Additionally, the study aims to explore the impact of stereo-related paradigms on SSVEP signals in the same execution mode.

**Figure 4:**
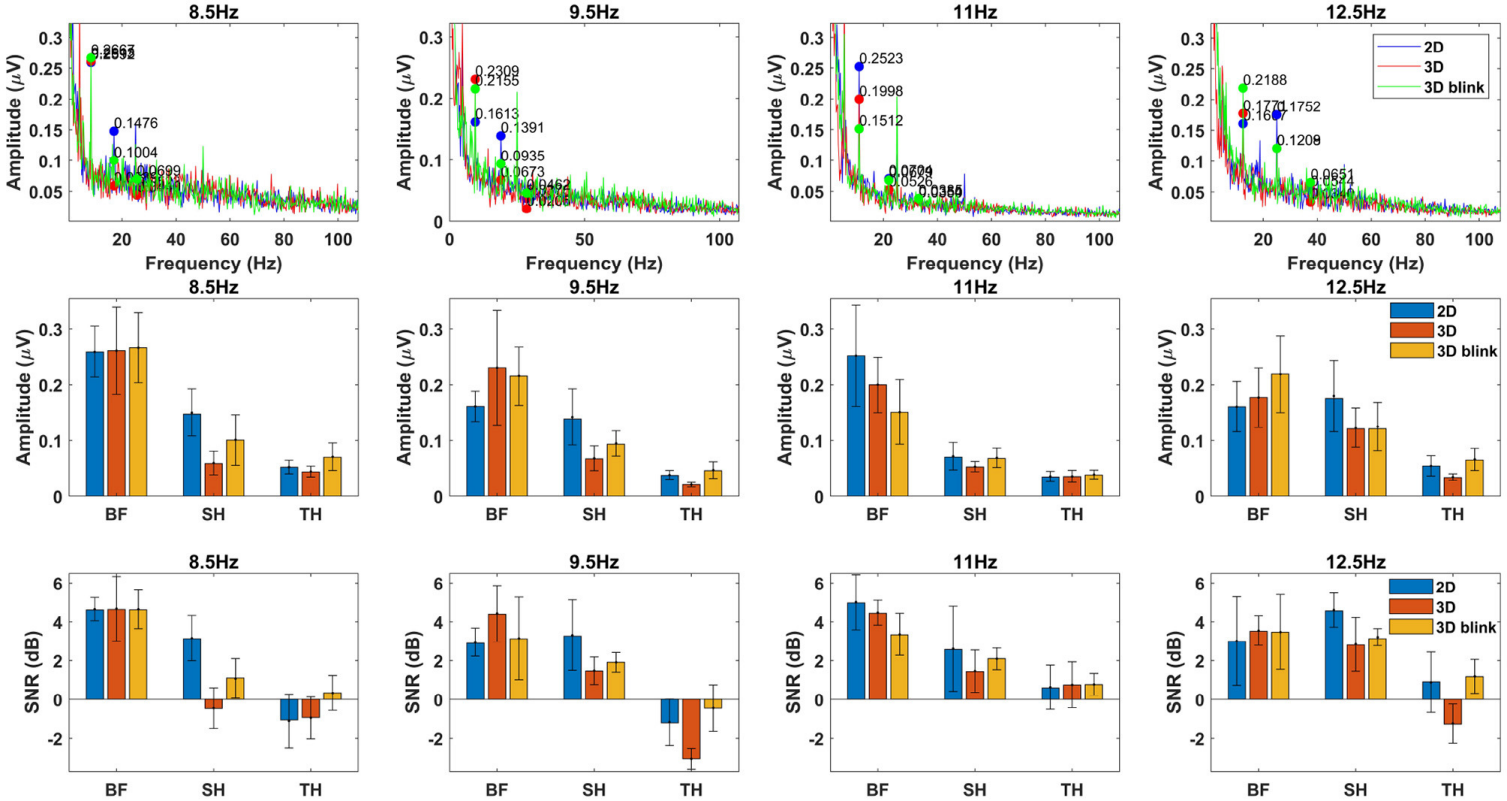
Feature comparison of four SSVEP targets. The first row shows the averaged amplitude spectrum of SSVEPs across subjects for all stimulus frequencies. The second row displays amplitudes of base frequency, second harmonic, and third harmonic calculated from the results, and the third row represents the SNRs computed. Here “BF” stands for base frequency, and “SH”, “TH” stands for the second and third harmonics of the target frequency. The EEG signals used for the analysis were obtained by averaging across trials for all subjects in the 2D, 3D, and 3D-Blink paradigms, and divided into four groups based on the different stimulus frequencies.

**Figure 5:**
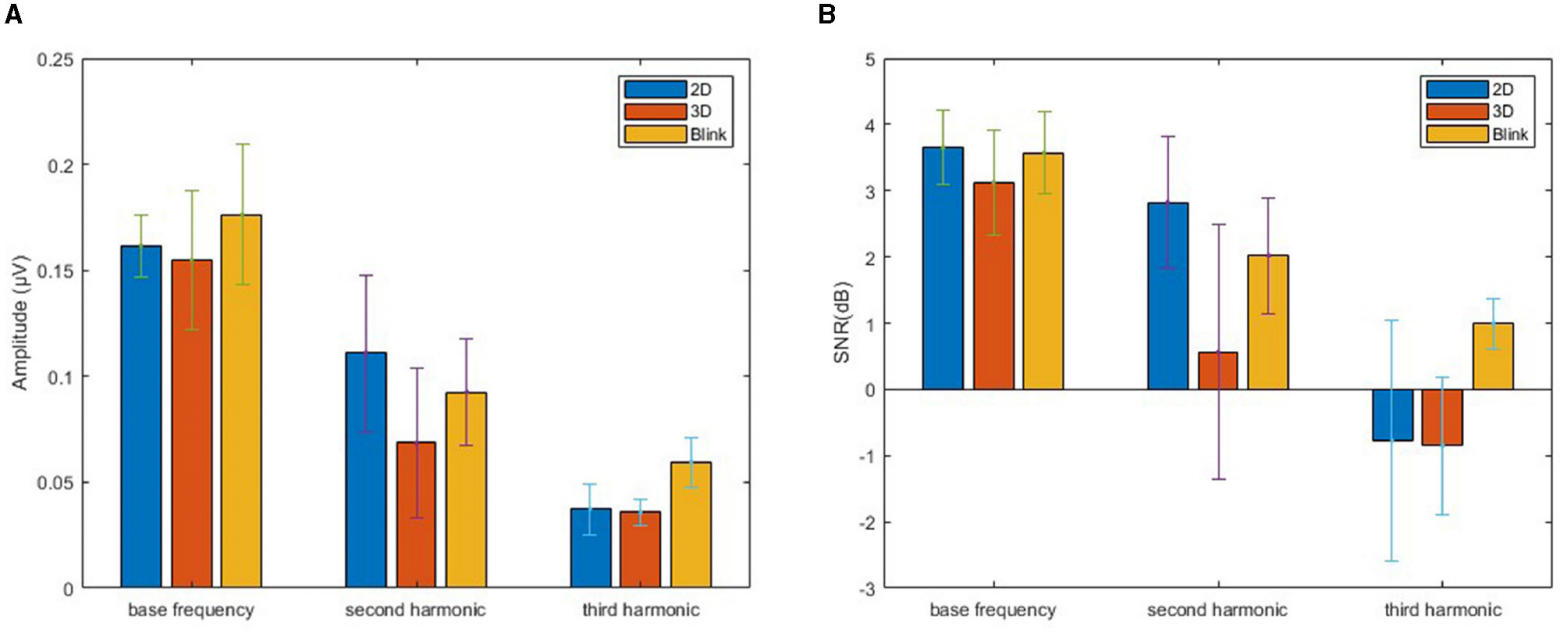
Amplitude spectrum and SNR. Bar chart obtained by averaging the results from all stimuli, with error bars, used to compare the differences in SSVEP features across different paradigms. **(A)** Amplitude. **(B)** SNR.

To achieve these objectives, we primarily focus on evaluating the performance differences based on the amplitude spectrum and SNR for the SSVEP signal under different paradigms, while combining the results of feature comparison and recognition performance analysis to explore their correlations. Figure 4 shows feature comparison of EEG data from all targets with four stimulus frequencies, which are grouped related to different stimuli presentation as 2D, 3D and 3D-Blink. The first row to the last row represent the cross-subject average amplitude spectrum marking the target frequency, the mean amplitudes of the fundamental frequency and harmonics, and the corresponding signal-to-noise ratio levels. By calculating the average values for different target stimuli, we derived the average amplitude and SNR depicted in Figure 5 to compare the overall levels of SSVEP features across different paradigms.

The recorded EEG data of 1,800 trials (10 participants × 60 trials × 3 tasks) and their corresponding event markers were grouped based on target frequency and task type, resulting in 12 sets of data (four targets × three tasks), each set containing 150 trials. We then computed the amplitude spectra and SNRs of the target frequencies based on these grouped data. By examining the amplitude spectra of all stimulus frequencies, clear peaks corresponding to each frequency were observed in Figure 4.

We conducted separate comparisons and analyzes of the characteristic differences among different frequency targets. This was done because the 3D binocular visual environment generated by VR differs from the flat screens traditionally used in the SSVEP experiments. Factors such as the participant's perspective changes during the experiment may indeed have some influence. However, in this experiment, there are certain differences in the background between the 2D and 3D stimuli. The experimental design aimed to highlight the immersive and stereoscopic effects of the 3D stimuli. However, as a result, the backgrounds of different targets in the 3D environment differ. Specifically, as shown in Figure 1A, the 3D target in the upper part has a pure sky background, while the backgrounds for the other three targets have a clearly extended floor visible in the distance.

In terms of results, we observed that the amplitude and SNR level for the left and right targets in the 2D paradigm were significantly better than those for the top and bottom targets. This suggests that there is a clear decrement in performance for 2D targets due to changes in perspective within the 3D space, while targets in the 3D paradigm exhibit better adaptability. There were no significant differences among the different targets in the 3D luminance paradigm. However, for the 3D-Blink paradigm, the first target with a flickering frequency of 8.5 Hz had a higher SNR compared to the subsequent three targets. Considering that our experiment was conducted sequentially in each trial, we speculate that the human eye may develop a certain tolerance to the visual stimulus of blinking, adapting to it over time and thus reducing its provocative nature. This may lead to a decrease in stimulation but an improvement in comfort.

Regarding the comparisons between different paradigms, we conducted an analysis combining the information from Figure 5 along with the results of Bonferroni corrected *post-hoc* pairwise comparisons using paired *t*-tests for different groups. The results indicate that there were no significant differences between the three paradigms in terms of base frequency (*p*>0.05). However, for the second and third harmonic frequencies, the 2D paradigm exhibited significant differences in SNR levels compared to both the 3D-Blink and 3D paradigms (*p* < 0.05). Additionally, despite the 3D-Blink paradigm having a lower base frequency SNR compared to the 2D paradigm, it showed superior SNR across the base frequency, second harmonic, and third harmonic levels when compared to the 3D paradigm.

We attribute this observation to the overall weaker stimulation intensity of non-luminance-modulated stimuli compared to luminance-modulated stimuli. Compared to 2D targets, the amplitude and SNR of the harmonics are lower for 3D targets. From an intuitive perspective, this is likely due to differences in the visual presentation of images between the 3D and 2D paradigms. The 3D targets constructed using Unity materials, despite being adjusted for brightness or transparency using the same methods and parameters as the 2D stimuli, exhibit more irregular gray scale distribution due to lighting and depth cues. Additionally, the distance from each point on the 3D sphere to the observer's retina varies due to spatial positioning. With planar stimuli, we can assume that the stimulation intensity is consistent for every point on the graphics, resulting in a uniform perception within a certain area for the observer. However, when stereoscopic targets are observed, the visual image generated exhibits non-uniform properties, which makes the fundamental frequency response still pronounced in the SSVEP results, while the harmonic components are suppressed.

### 3.2 Recognition performance

We demonstrated the significant difference between 2D and 3D stimuli through the accuracy of classification performance and ITR. This finding reflects and contrasts the most direct and fundamental ability of the VEP-based BCI to perform classification tasks.

**Figure 6:**
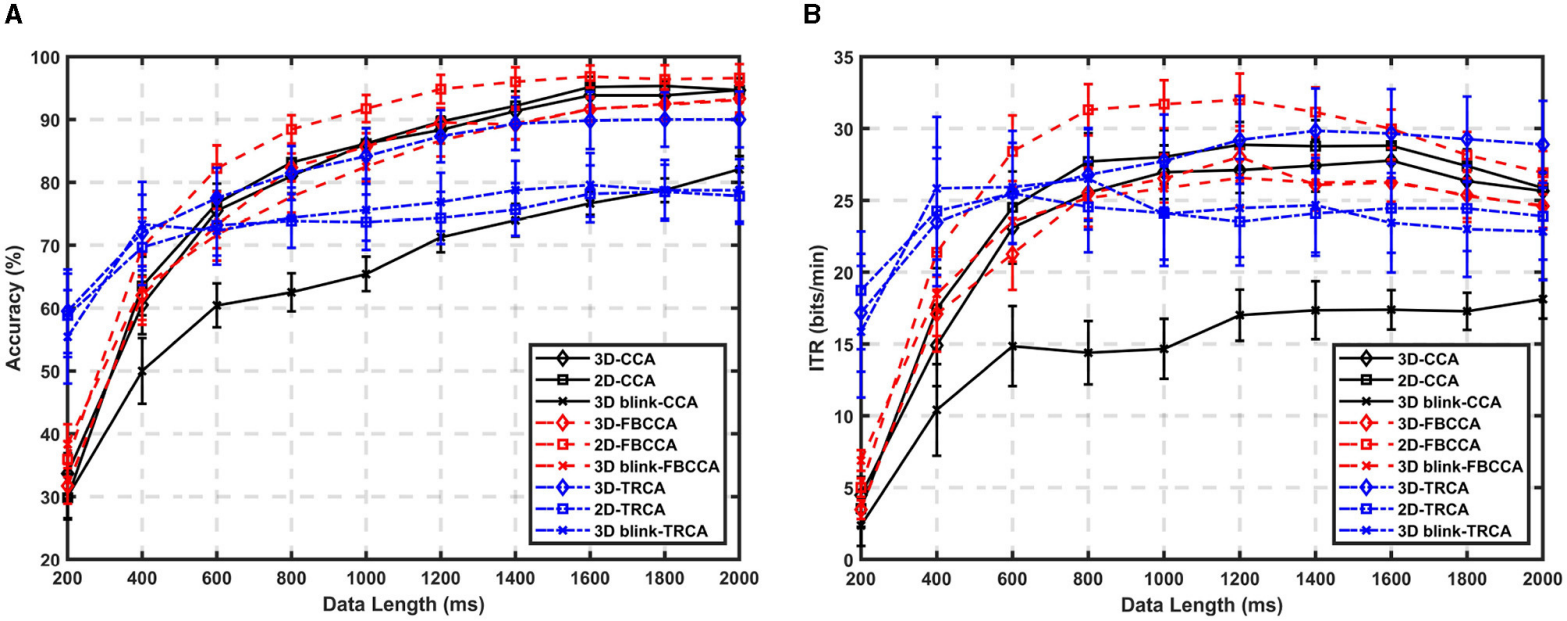
Comparison of offline performance during target selection task between the CCA, FBCCA, and TRCA methods when using the data from experiments showing 2D, 3D and “3D-Blink” paradigms in VR scene. **(A)** Averaged target identification accuracy across subjects. **(B)** Averaged ITRs across subjects.

The ratio of correct identifications to the total number of trials in a task is considered the classification accuracy. We calculated the accuracies for three tasks with three different algorithm. Figure 6 displays the variation of classification accuracy with data length for 10 participants using CCA, FBCCA, and TRCA methods. The experimental data are marked based on the onset time of the target flickering in the experimental procedure, taking into account a visual delay of 130 ms during processing. A complete trial has a data length of 4 s, and for offline analysis, we used data lengths ranging from 0.2 to 2 s with intervals of 0.2 s for classification. The target with the highest correlation coefficient is considered the identified target. The classification accuracy was calculated using leave-one-out cross-validation, which divides the collected data into 60÷4 = 15 sets, where 60 represents the total number of trials for a single task and 4 represents the number of targets. For each set of data, the overall accuracy and ITR of the remaining 14 sets of data were calculated, and the average accuracy and ITR from each calculation yielded the final accuracy and ITR.

As shown in Figure 6A, the best average accuracy for the 3D paradigm was 85.67% and for the 2D paradigm it was 91.73% with a data length of 1 s and the FBCCA method. However, there was little difference in accuracy between the two paradigms when the data length was extended to 2 s.

Based on the observations from the results in Figure 6, we can see that when comparing different algorithm groups, there is no significant difference in the improvement trend of accuracy between the 2D and 3D paradigms in CCA (*p*>0.05). 3D paradigm slightly underperformed compared to the 2D paradigm, while the performance of the 3D-Blink paradigm in CCA was significantly lower than the two luminance-modulated stimuli.

As there are no significant differences in classification accuracy between 3D targets and 2D targets when controlling variables and displaying them in a VR environment, we can conclude that the effect of stereoscopic perception is limited in the context of conventional SSVEP paradigms. For the SSVEP paradigm, there may be a certain conflict between visual evoked potentials and cognitive activities. On the basis of the known results, it is believed that the perceived stereoscopic effect cannot directly provide usable gains for traditional SSVEP.

In the case of FBCCA, we set FBCCA to use three sub-bands and generate up to three harmonics for the reference signal, and observed a significant advantage in the performance of the 2D paradigm compared to the 3D paradigm. The average accuracy of the 2D paradigm increased by 5.56% with a 1-second data length, while the 3D paradigm increased only by 0.50%. We can clearly observe that results of the 2D paradigm show a more pronounced increase in accuracy as data length increases, especially in the 0.6–1 s interval. This result aligns with the analysis of the harmonic component SNR, which was found to be lower for the 3D target compared to the 2D target. The FBCCA method further divides the SSVEP signals into multiple sub-bands using filter banks, which means that the performance improvement of FBCCA relies on extracting more information from the analysis of harmonic components.

The TRCA algorithm has shown significant superiority over CCA and FBCCA in terms of recognition performance, especially when the length of the data is short. However, in this experiment, as the length of the data used increased, there was no significant improvement in performance. Even with a data length of 2 s, all three paradigms achieved performance below an average of 90%. The 3D luminance paradigm demonstrated the highest recognition performance, followed by the 3D-Blink paradigm, while the 2D paradigm using planar stimuli showed the lowest performance. Although efforts were made to address time synchronization issues in the VR-BCI system, small errors or accumulated errors may still exist due to animation implementation and visual stimulus display instability, leading to phase shifts and inconsistent event labeling. These factors probably contributed to the limited performance improvement of the TRCA algorithm with longer data lengths. Overall, except for the 0.2–0.6 s interval with relatively good performance, the ITR levels were generally lower than those achieved by the FBCCA method.

**Figure 7:**
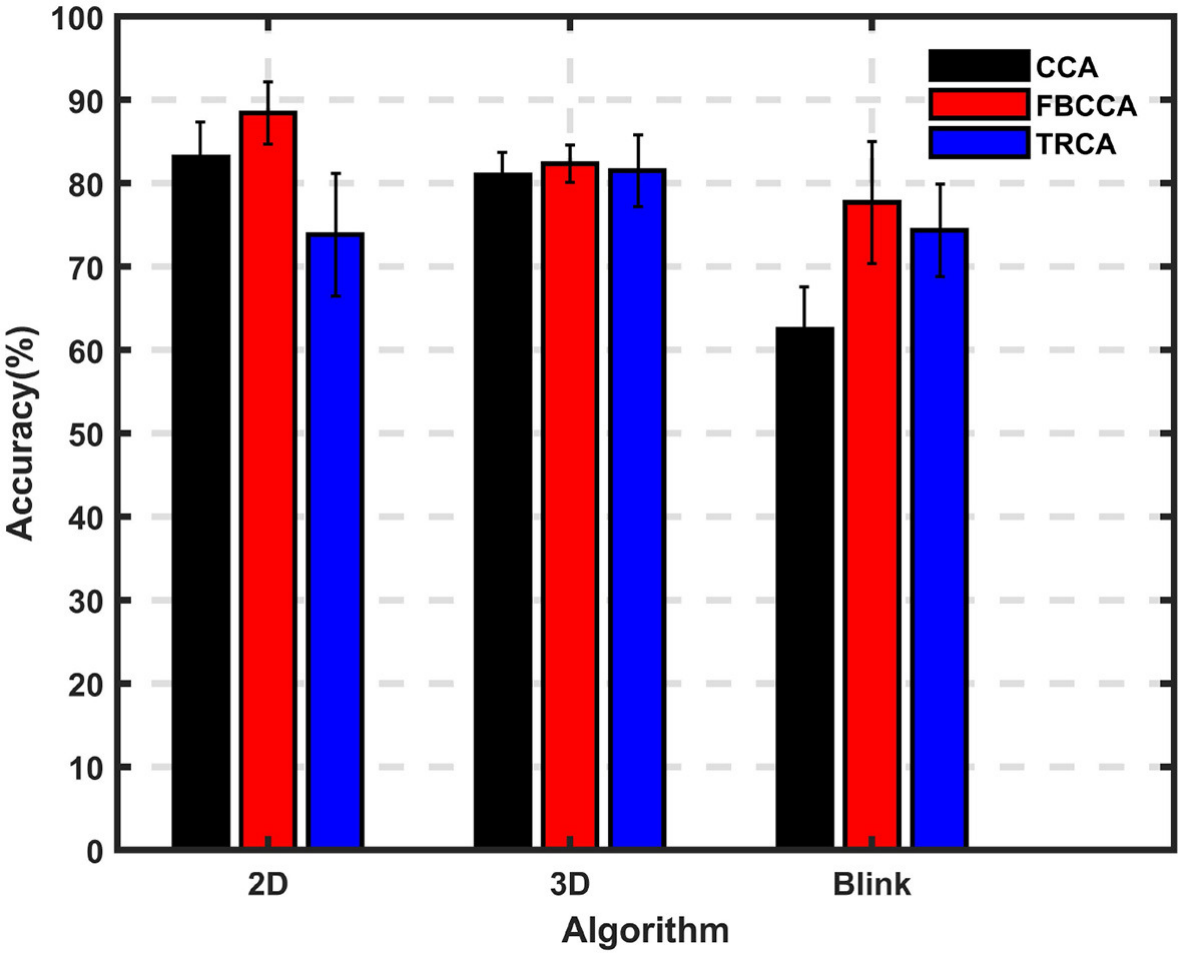
Average accuracy of different algorithms for different paradigms. Performance of three algorithms (CCA, FBCCA, TRCA) for three paradigms (2D, 3D, 3D-Blink) when using a data length of 1 s.

Figure 7 compares the recognition accuracy of different paradigms using different algorithms, with a data length of 1 s. The results of this comparison lead to the following conclusions: (1) Under the CCA algorithm, there is little difference in performance between the 2D and 3D luminance modulation paradigms, indicating that their recognition performance is comparable. However, the 3D-Blink paradigm performs worse due to its lower response magnitude at the target frequency. (2) The FBCCA algorithm shows greater improvement for the 2D and 3D-Blink paradigms, as it effectively utilizes harmonic information. However, for the 3D paradigm, the gain is minimal due to the lower signal-to-noise ratio of the harmonic components. (3) Under the TRCA algorithm, both paradigms of 3D target exhibit some performance advantages, suggesting that TRCA can be beneficial for these paradigms.

**Figure 8:**
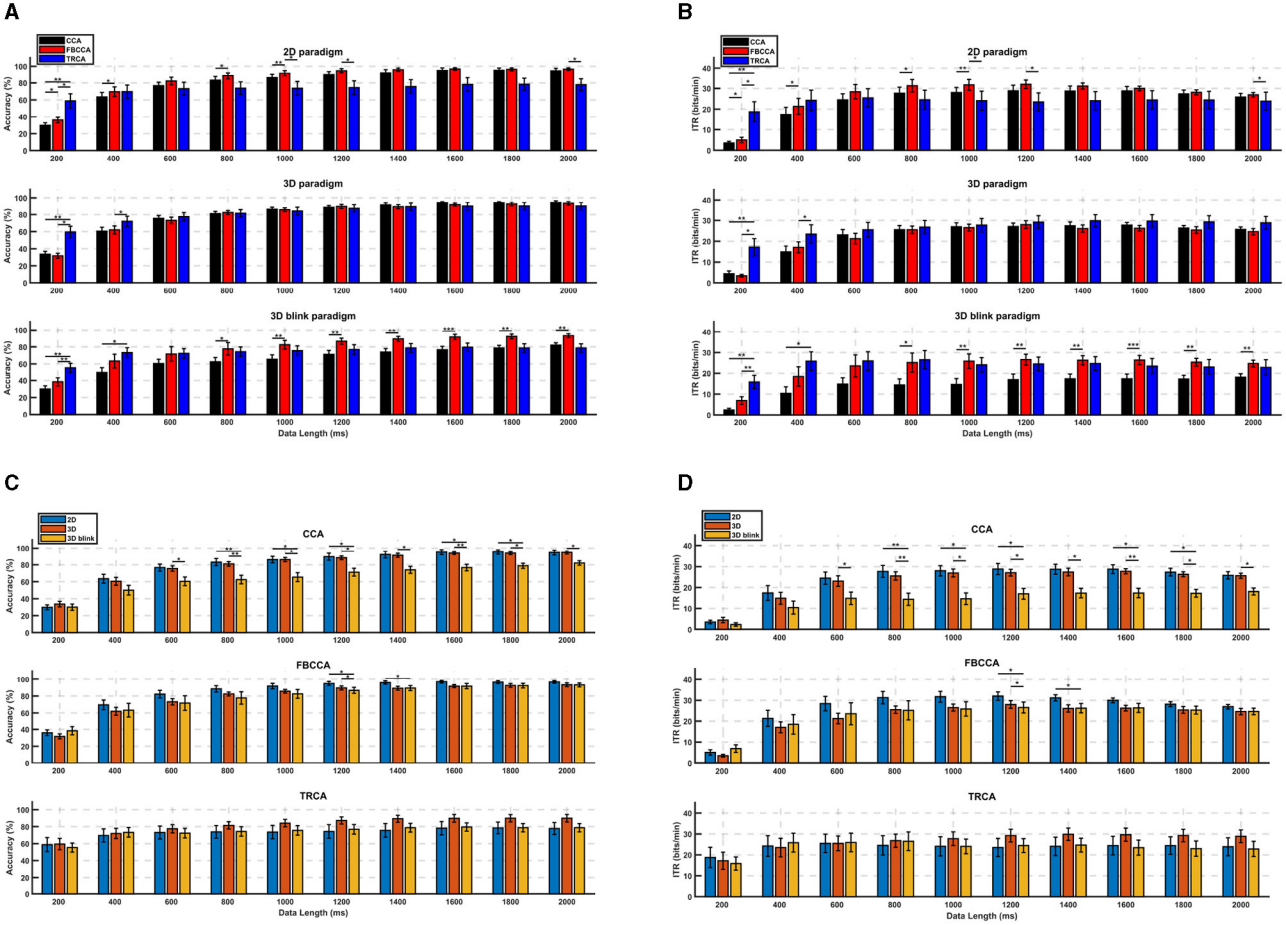
Performance comparison of different algorithms and different paradigms from the offline BCI experiment. **(A)** Average classification accuracy, **(B)** mean ITRs across subjects of different paradigms. **(C)** Average classification accuracy, **(D)** mean ITRs across subjects different algorithms. The error bars indicate standard errors. The asterisks indicate the significance of paired *t*-tests (*p < 0.05, **p < 0.01, ***p < 0.001).

Figure 8 displays bar charts with error bars to illustrate the performance differences and the significance of these differences for various paradigms and algorithms across each time length. Overall, the results indicate that the performance of the 3D paradigm remains relatively consistent across the three algorithms, whereas the 3D-Blink paradigm shows a notable improvement when using the FBCCA algorithm compared to CCA with data length of 0.6–2 s. When comparing different paradigms for each algorithm, it is evident that under the CCA algorithm, the 2D and 3D paradigms often outperform the 3D-Blink paradigm. On the other hand, with the FBCCA algorithm, the performance of the three paradigms tends to converge as the time length increases. Notably, the TRCA algorithm does not display a significant overall performance trend change across the three paradigms. These findings suggest that the 3D-Blink paradigm benefits more from the FBCCA algorithm than the 3D paradigm and can achieve performance levels similar to the 2D paradigm.

### 3.3 Questionnaire feedback

The subjective feedback results are shown in Table 1, which contains the comfort level scores of the 2D, 3D, and 3D-Blink paradigms from the questionnaire provided to subjects after the experiments. Repeated-measures analysis of variance (ANOVA) was employed to assess the significant differences between the three paradigms in comfortable experience, the results $\left[F_{\left(2,27\right)}=27.86,\;p<10^{-5}\right]$ of which indicate that subjective comfort level scores differ significantly between the 2D, 3D, and 3D-Blink paradigms. It can be observed from comparison of the mean value of comfort level score that the 3D-Blink paradigm has the highest level of comfort, followed by the 3D luminance stimulation paradigm, and the 2D paradigm has the lowest level of comfort. VR scores are derived from a comprehensive assessment of factors including visual clarity, presence of 3D motion sickness, comfort of wearing, fatigue during the experiment, and overall experience. The average result to some extent indicates that participants are still influenced by certain discomfort factors associated with the use of VR.

**Table 1:** Subjective comfort questionnaire on visual feeling for different paradigms and overall VR user experience.

	**Scores (ranges from 1 to 10)**			
**Subject**	**2D**	**3D**	**3D-Blink**	**VR**
S1	3	9	6	8
S2	1	6	10	9
S3	4	6	8	6
S4	7	9	9	9
S5	5	7	10	8
S6	2	5	8	7
S7	3	6	9	6
S8	2	7	10	8
S9	1	4	7	4
S10	3	5	8	5
Mean	3.1	6.4	8.5	7.0

Scores ranges from 1 to 10, where 1 indicates totally unacceptable, and 10 indicates very comfortable.

## 4 Discussion

This study utilized VR, a binocular display device, to construct an SSVEP-BCI paradigm, considering the stereoscopic or depth attributes of the stimulus targets. Two new paradigms were proposed in this study: the 3D paradigm and the 3D-Blink paradigm, which were investigated along with the traditional 2D planar paradigm. The results demonstrate that there were almost no differences in recognition performance between the 2D and 3D paradigms in static selection tasks when using the CCA method, but a significant difference was shown in frequency feature analysis. The lower signal-to-noise ratio and limited performance optimization obtained through the FBCCA method in the 3D paradigm suggest that there is still room for improvement in utilizing this new paradigm in VR-BCI systems. This study demonstrates the feasibility of implementing the proposed new paradigms in VR-BCI systems and the fact that stereoscopic vision significantly affects SSVEP features, highlighting the need for further research to investigate the sources of these differences and explore potential optimization strategies.

### 4.1 System of VR-BCI and implementation of VR-SSVEP

Our study aims to compare the effects of presenting 2D and 3D targets in VR stereoscopic vision on SSVEP. Initially, we planned to compare 2D targets on a flat screen with 3D targets in VR. However, we found that this comparison method introduced many uncontrollable factors, such as screen brightness, viewing angle, and participants' perception of the actual size of the stimuli, due to differences in display principles. Finally, we chose to present all 2D and 3D stimuli in a VR environment to ensure a fair comparison under the same conditions. To maintain visual consistency with traditional PC-SSVEP visual presentation on flat screens, we added a flat gray background for the 2D paradigm. This eliminates the influence of depth information, allowing us to obtain purer 2D visual stimuli and avoid introducing stereoscopic features that may arise from the inclination of 2D targets or depth contrast with the background. In other words, we simulated the presentation of 2D stimuli on a flat screen and 3D spherical stimuli in the same spatial location in a VR environment. All stimuli used in the experiment were circular or spherical in shape to avoid the potential influences of the stimulus area and optical distortions at the edges of VR displays (Duan et al., 2022). Additionally, it should be noted that the limited field of view in VR leads to visual distortion of non-currently aiming stimuli that flicker in the peripheral vision. This effect is equitable when applied to both 2D and 3D paradigms and can be considered a characteristic of VR vision without causing additional system differences.

An important factor for normal display and comfortable use of VR is the alignment between the user's IPD and device settings, which could exacerbate visual fatigue or 3D vertigo (Mun et al., 2012). In this study, due to the lack of automatic adjustment capabilities in the device, we simply used a fixed IPD setting in our experimental setup. As there were few reports of 3D dizziness or significant fatigue from the majority of participants, while the duration of the experiment was not very long, we believe that the influence of IPD variations can be neglected in the analysis of the results.

Some users reported fatigue and dizziness associated with wearing VR headsets, which could potentially impact the experiment. To address this issue, we implemented session-block separation, allowing participants to rest and return to their natural visual state between sessions. Additionally, we further divided sessions into blocks with short breaks, creating an overall relaxed experimental procedure. Although we believe that these measures effectively controlled for this factor, questionnaire feedback indicated some residual impact.

The VR-BCI system implemented in the study did not prioritize implementation of the mobile system, but the display parameters and graphic performance of the VR headset may have influenced the results. By adjusting system design and software parameters, we have successfully reduced the error in event marking to an acceptable range. With the 1 ms graphics engine update interval, we believe that the influence of this factor on TRCA synchronization can be effectively mitigated.

**Figure 9:**
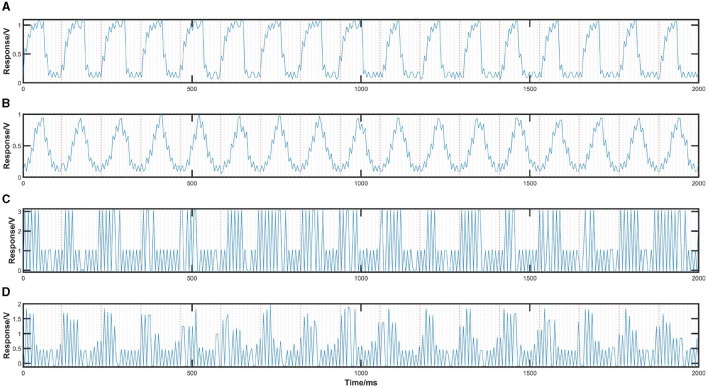
Results of validation tests using TSL257-LF. **(A)** 2D-flip displayed by PC-LCD, **(B)** 2D-sin displayed by PC-LCD, **(C)** 2D-flip displayed by VR-HMD, **(D)** 2D-sin displayed by VR-HMD.

TRCA's performance remains below anticipated levels, potentially attributed to phase shifts arising from the refresh mechanisms of VR in simulating SSVEP stimuli, suggesting a significant underlying issue. We investigated the display mechanisms of VR-HMD and traditional desktop LCD monitors through the TSL257-LF sensor, particularly focusing on their refresh strategies and the modulation of luminance, aiming to validate whether the simulation process of the stimuli meets expectations. Figure 9 presents stimuli in both flip and sin modes at 8.5 Hz, displayed using desktop LCD monitors and VR-HMD, all rendered by Unity. Comparing Figures 9B, D, we can observe the differences between the VR-HMD and desktop LCD monitors, where VR-SSVEP luminance modulation is achieved by an 8.5 Hz sinusoidal wave carried by a 90 Hz square wave, with the 90 Hz carrier for left and right eye displays having opposite phases. Upon further investigation, we understand that this is attributed to VR's adoption of black frame insertion (BFI) technology (Kurita, 2001; Hong et al., 2005), which is utilized to enhance visual clarity, reduce motion blur, and improve user comfort by integrating black frames between image frames, thus aligning the display refreshment more closely with the natural human vision process. In terms of temporal features, the waveform in Figure 9D lacks the same level of stability and regularity observed in the PC response shown in Figure 9B. While certain parts exhibit consistent 90 Hz changes, highlighting the characteristics of the high-frequency carrier, anomalies occur in other segments, potentially due to system delays or other factors leading to temporary non-responsiveness. Following these interruptions, the carrier continues to oscillate at a 90 Hz frequency, albeit with a shifted phase due to these unexpected pauses. This discontinuity not only disrupts the carrier's coherence but also introduces phase interference to the 8.5 Hz sinusoidal wave, impacting the overall waveform's uniformity and stability. Although frequency domain analysis and results from other algorithms suggest that VR-SSVEP frequency properties are relatively stable, the impact on temporal features may indeed be the primary reason for the limitations in TRCA performance. Adapting to the refresh method of VR devices and optimizing VR-BCI systems accordingly will be an important research direction in the future.

Ma et al. (2018) implemented a VR-BCI system in a 40-target SSVEP experiment with a 300-millisecond data length, achieving an accuracy of 82%. The study showed that SSVEP signals collected in VR-BCI settings effectively utilized TRCA classification. However, both this study and Ke et al. (2020) point to suboptimal performance of TRCA in VR or AR combined with BCI systems. It is suggested that display performance, temporal precision of event markers, and stimulus implementation logic should be reconsidered and improved to ensure temporal stability of stimulus presentation for addressing synchronization issues. Additionally, a wider variety of training-based methods may be considered for SSVEP-BCI under VR/AR conditions to provide robust, high-performance classification algorithms tailored for these scenarios. Another way to improve synchronization in various scenarios is to modify the TRCA algorithm. Expanding the data extraction window beyond the reference event interval can help when trial-to-trial latency jitter makes it difficult to identify task-related components (TRCs). By accounting for latency jitter in each TRC within this extended range and incorporating this into the TRCA algorithm, it may adapt and perform better in situations with imperfect synchronization, allowing for the identification of optimal reproducible components.

### 4.2 Impact of stereo-related mechanisms

When discussing the impact of VR environments on EEG activity, it is important to understand their sources. On the one hand, stereo and depth perception can elicit ERP features, as shown in previous studies (Guo et al., 2022). However, these features are difficult to utilize in SSVEP-BCI systems and may be suppressed by sustained stimulation. In our study, prolonged or high-frequency stimuli appeared to make participants familiar or adapted to fixating on the current target, which benefits short-term TRCA methods but not long-term CCA-based methods. Although VR presents 3D objects through binocular disparity, depth cues are still necessary. Specifically, 3D objects rendered by the program may have uneven grayscale due to material and lighting effects, resulting in differences from 2D targets perceived by the human eye. The perception of distance and stereopsis in space is synthesized in the brain on the basis of binocular disparity. These differences in image presentation might affect the SSVEP features.

In our study, the SNR of the harmonic components for the 3D targets was much lower compared to the uniform 2D targets. This phenomenon rendered methods such as FBCCA, which rely on the extraction of harmonic components, ineffective. However, it may benefit multi-target SSVEP encoding by reducing harmonic interference with other targets' base frequency, thus improving overall system SNR and encoding capability. Liang et al. (2021) have shown effects similar to the attenuation of harmonics, but with stimuli placed in the left and right visual fields with the screen display. Moreover, experiments involving binocular rivalry (Yue et al., 2020) have also raised the issue of differences in SSVEP response phase patterns between the left and right visual fields. Similar effects may exist in binocular displays compared to natural visual displays, causing phase shifts in SSVEP due to differences between left and right visual perceptions. Specific research is needed into binocular VEP's temporal and spectral differences under stereoscopic vision and optimization of spatial channel deployment and phase modulation for the source stimuli.

Within the framework of an overt paradigm, our hypothesis posits that there are distinct physiological differences in the processing of binocular image synthesis between 2D and 3D stimuli. Two-dimensional stimuli in space are also perceived binocularly, but their planar nature lacks the third-dimensional attribute for stereoscopic perception, leading to only slight positional differences in the left and right visual fields and resulting in the classic features of SSVEP due to their uniform characteristics. In contrast, 3D stimuli, like the sphere in our study, show depth variations when observed directly, such as the top of the sphere facing the viewer. The depth perception inherent in 3D stimuli, achieved through the fusion of binocular images with left-right disparities, may introduce phase shifts in the conduction of SSVEPs in the nervous system, resulting in differing conduction patterns and reduced harmonic energy for 3D stimuli. However, the fundamental frequency energy may remain relatively unaffected compared to 2D stimuli. These theoretical propositions warrant further investigation, especially regarding the impact of varying depths in VR-supported environments on SSVEP characteristics.

The Blink mode of the 3D paradigm has received positive feedback in terms of user comfort, but overall it has lower accuracy compared to the conventional luminance modulation SSVEP, whether it is for 3D or 2D targets. Considering that the 3D-Blink paradigm actually uses an inversion of opacity to transition the stimulus state, the decrease in SNR in this case may be attributed to the insufficient contrast between the purely default material white solid and the similarly light-colored background. Whether factors such as color or lighting materials have an impact on this phenomenon requires further research to provide a conclusive explanation.

Additionally, the 3D-Blink paradigm utilized in this study induces SSVEP in the flip mode, which operates through alternating states of 0% and 100% transparency. Similar to SSVEP based on luminance modulation, the sin mode of the 3D-Blink paradigm may yield better or different SSVEP responses, which needs further exploration in future research.

In the 3D-Blink paradigm, the presentation creates a persistence of vision, forming a semi-transparent spherical target for the observer. From the experiments, we also discovered that participants could stably perceive the background behind the target, such as the floor or the distant horizon of the VR space. This suggests that by incorporating focal cues before, after, and in the center of the stimuli, the 3D-Blink paradigm allows users to expand the visual state induced by the same stimulus target. This mechanism, which operates in a depth-related manner, may offer a novel approach to enhance the encoding capabilities of VR-SSVEP. This could also display additional information, allowing users to switch between SSVEP targets using peripheral vision or focused attention. At the same time, users can extract other information from the scene through their gaze, thereby enhancing the interactive capabilities of VR-BCI systems.

**Figure 10:**
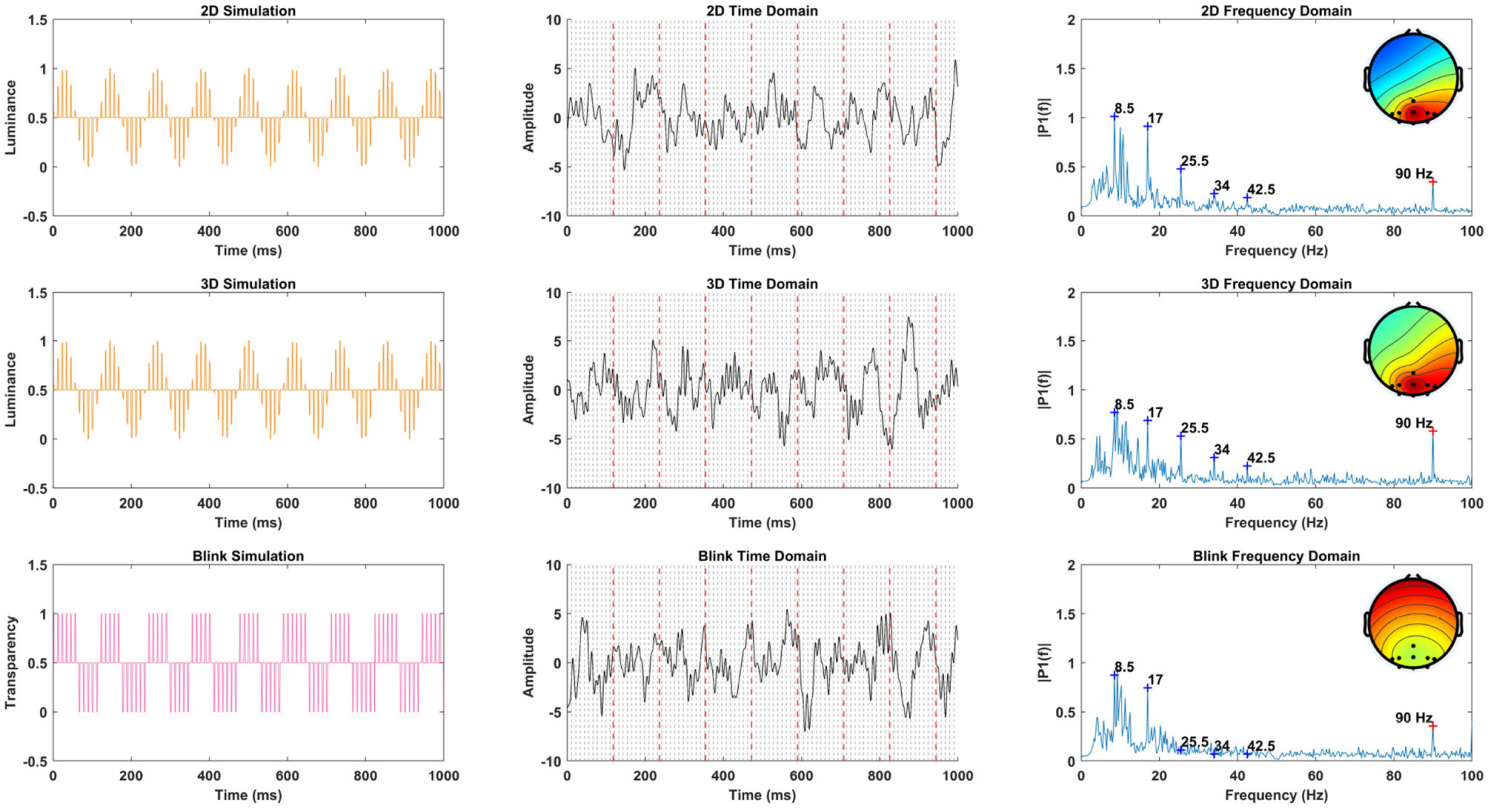
Comparative Analysis of SSVEP characteristics of three paradigms.

In this study, the proposed paradigms provide diverse visual spatial information, potentially influencing the phase and spatial distribution of SSVEP responses, warranting a detailed analysis of SSVEP characteristics. Our comparative analysis of SSVEP characteristics across the 2D, 3D, and 3D-Blink paradigms, especially focusing on Subject 2's data, is illustrated in Figure 10. The first column of Figure 10 showcases simulated SSVEP stimuli for 2D and 3D visual conditions, incorporating a depiction of transparency variations in the 3D-Blink paradigm. The second column captures the brain's response to these stimuli, represented through averaged EEG time series, with red dashed vertical lines indicating the 8.5 Hz frequency and gray dashed lines accentuating the 90 Hz frequency. The third column presents the power spectral density (PSD) analysis, where blue crosses identify target frequencies and their harmonics, and a red cross highlights the prominent 90 Hz peak, along with topographic maps that illustrate the spatial distribution of EEG responses.

Feedback from the time series not only aligns closely with the 8.5 Hz trend but also exhibits components of 90 Hz, reflecting the influence of VR display mechanisms mentioned in the previous section. Apart from the target frequency of 8.5 Hz and its harmonic components, the PSD plots prominently feature a peak at 90 Hz. The spatial distribution of PSD energy for the 2D and 3D paradigms appears similar, consistent with the expected SSVEP spatial propagation results considering the 8.5 Hz stimulus was positioned to the participant's left. The 3D-Blink paradigm's topographic map distribution is more symmetrical and exhibits weaker intensity compared to the 2D and 3D paradigms.

**Figure 11:**
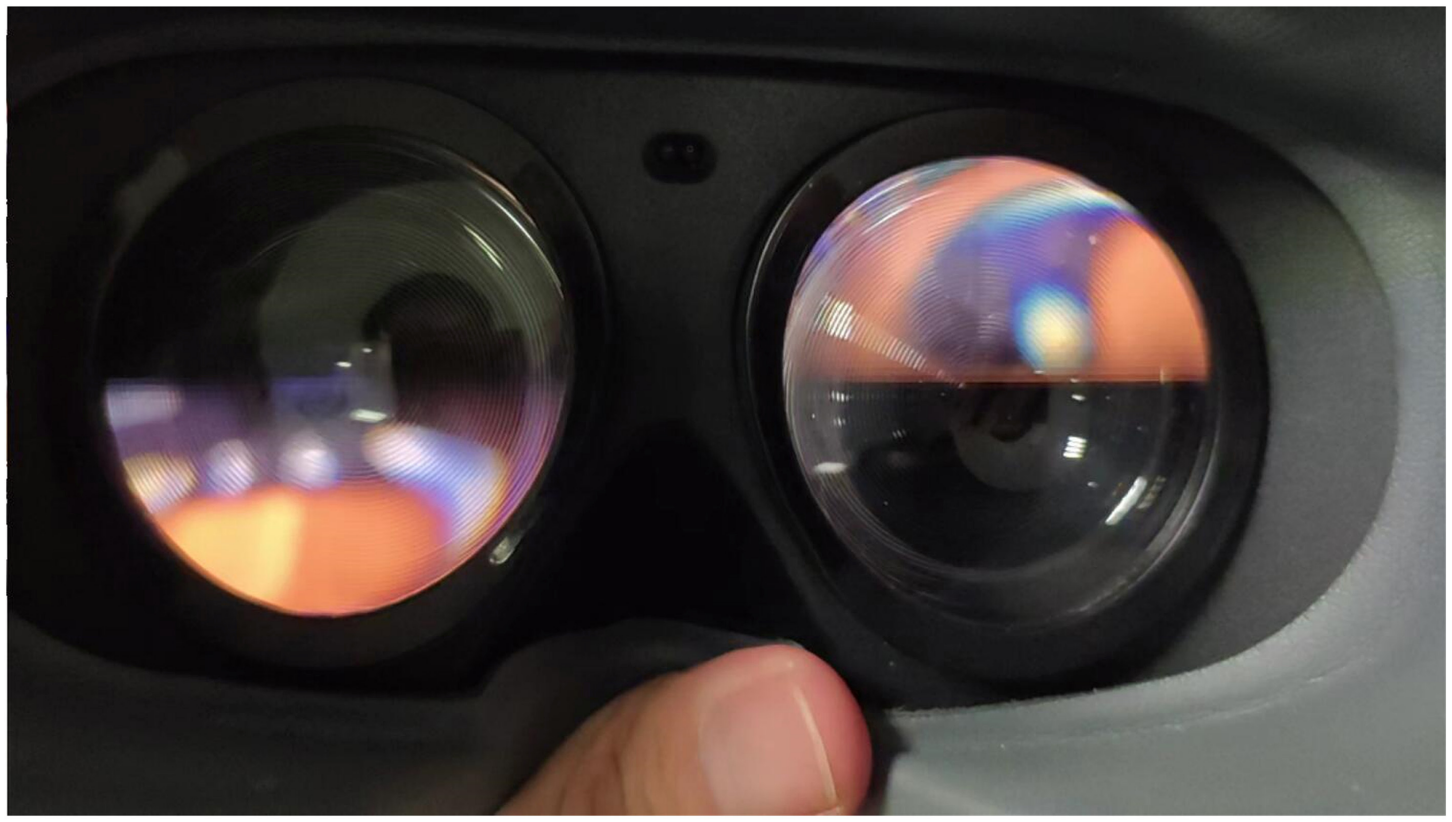
One frame of VR binocular display captured at 1/1,000 shutter speed.

Furthermore, the foundation of depth perception relies on binocular display. The characteristics arising from the dual-screen refresh mechanism in VR-SSVEP are noteworthy. In our study, the originally simulated 8.5 Hz stimulus effectively modulates atop a 90 Hz square wave, with the phase of the 90 Hz square waves for the left and right eyes inverted. As shown in the Figure 11 captured with a smartphone camera at a shutter speed of 1/1,000, the phenomenon of black frame insertion in VR displays is observable, along with the complementary characteristics of the left and right displays. Although their combination should yield an SSVEP stimulus light intensity change response identical to that of an LCD display, physically, each eye indeed receives the 8.5 Hz stimulus on a differently phased 90 Hz carrier. Research indicates that the anatomical outcomes of the visual neural pathway are essentially symmetrical, implying the contributions from the left and right eyes toward the EEG response generated in the occipital area for SSVEP are ultimately equivalent (Wu and Wu, 2017). However, this pertains to continuous identical stimuli; the PWM refresh method with dual-screen utilized by VR might introduce other impacts. Future research could explore the effects of binocular display's refresh mechanisms on SSVEP-BCI and refine algorithms to accommodate these mechanisms, enhancing the efficacy and adaptability of SSVEP-based VR-BCI systems.

### 4.3 Significance, limitation, and future directions

Deploying BCI technology in immersive VR applications of today inevitably encounters the influence of perspective and distance in the 3D space. In addition to accuracy and response speed, user comfort during repeated usage is a crucial factor of concern. Consequently, exploring the impact of 3D target presentation mechanisms and depth-related visual cues on VEP-BCI is of significant importance. The findings of this research primarily uncover the differences between the 3D and 2D paradigms in the characteristics of SSVEP, as well as the availability and potential of new paradigms. The non-significant performance difference between the 3D and 2D paradigms has a positive implication for diversified VR-BCI interaction design. However, the discrepancies in the optimization methods resulting from feature differences indicate the need to further explore the stimulus modulation methods or algorithms in VR-SSVEP.

The main limitation of our study lies in the analysis of differences in features between the proposed 3D, 3D-Blink paradigms and the baseline 2D paradigm, which was limited to the frequency domain. Differences in features in the frequency domain may be caused by multiple factors. Our experimental design did not consider phase or time latency issues, and electrode setup was limited to traditional occipital regions, providing little insight from an EEG Montage perspective. Future studies should attempt to introduce channels in the temporal lobe region and obtain responses to monocular and binocular SSVEP stimuli at different spatial locations under the same conditions. The results of monocular and binocular responses could be compared, or the effect of recognition could be observed by setting different phase offsets for the stimuli. In future research, it will also be crucial to include a broader range of participants, particularly adolescents and the elderly, in order to extend the applicability and depth of the findings.

To further leverage the effects of stereoscopic perception in enhancing VEP-BCI, presenting IVEP or SSMVEP in a VR environment may be a better choice, where techniques such as spatial flipping actions, stereoscopic contraction/expansion transformations, rotation of textured surfaces in 3D, and changes in distance could potentially be employed to yield notable ERP responses for recognition. Last but not least, note that the full potential of TRCA has not yet been demonstrated on platforms like VR-BCI. Future studies should focus on improving the stability of visual presentation and system event synchronization while also exploring whether the presentation of stereoscopic target stimuli can provide TRCA with task-relevant components that exhibit good recognition and reproducibility.

Eye-tracking integration in VR devices is also considered an innovative and efficient interaction method. While this study does not directly compare the ITRs between VR headsets with eye-tracking and VR-BCI using SSVEP, it contends that even in the presence of eye-tracking technology, VR-BCI based on SSVEP still holds certain advantages and value. Eye-tracking relies on monitoring physical eye movements for information gathering, but human gaze is less reliable than a mechanical system due to subconscious movements, tremors, and response delays. Calibration failures in eye-tracking can occur due to head movements and squinting, which, in contrast, have a lesser impact on the reliability of EEG-BCI systems. Many systems address the Midas Touch problem (Jacob, 1991) by integrating eye-tracking with alternative inputs like gestures, showing the potential for BCI technology to complement eye-tracking. BCI can offer rich cognitive information and, theoretically, does not conflict with eye-tracking technology; their combination can enhance the diversity and efficiency of interactive systems. From a user experience perspective, relying solely on BCI or eye-tracking for interaction can be burdensome. However, exploring the integration of the SSVEP-BCI paradigm with eye-tracking indicates that combining these technologies could provide a superior interaction option, thereby enhancing the overall user experience. Additionally, this integration might further increase the overall ITR, paving the way for more effective and efficient composite interaction methodologies in the future.

## 5 Conclusion

As a comparative study, we compared the differences between 2D and 3D paradigms in a virtual reality (VR) based SSVEP multi-target selection task. Additionally, a new non-luminance-modulated visual stimulus method was proposed. The results showed that, under well-controlled variable conditions, there was almost no difference in recognition performance between the 2D and 3D paradigms in static selection tasks. However, certain factors inherent to VR may introduce slight performance degradation. The 3D-Blink paradigm, inspired by the 3D paradigm, further improved the comfort of visual stimulation, which exhibited significant performance degradation in non-trained algorithms, while maintaining usable recognition ability in trained algorithms. This study explored the use of stereo-related stimulation in the SSVEP paradigm, offering a new approach for developing user-friendly wearable EEG signal applications.

## Data availability statement

The raw data supporting the conclusions of this article will be made available by the authors, without undue reservation.

## Ethics statement

The studies involving humans were approved by the Ethics Committee of ShanghaiTech University. The studies were conducted in accordance with the local legislation and institutional requirements. The participants provided their written informed consent to participate in this study.

## Author contributions

HL: Data curation, Formal analysis, Investigation, Methodology, Software, Visualization, Writing – original draft, Writing – review & editing, Conceptualization, Project administration, Validation. ZW: Conceptualization, Formal analysis, Investigation, Methodology, Resources, Supervision, Writing – original draft, Writing – review & editing. RL: Formal analysis, Investigation, Writing – review & editing. XZ: Conceptualization, Resources, Writing – review & editing, Formal analysis, Methodology. TX: Resources, Conceptualization, Formal analysis, Writing – review & editing. TZ: Formal analysis, Resources, Writing – review & editing. HH: Conceptualization, Supervision, Writing – review & editing.

## References

[B1] Allison B. Z. Brunner C. Altstätter C. Wagner I. C. Grissmann S. Neuper C. (2012). A hybrid ERD/SSVEP BCI for continuous simultaneous two dimensional cursor control. J. Neurosci. Methods 209, 299–307. 10.1016/j.jneumeth.2012.06.022 22771715

[B2] Anzai A. Ohzawa I. Freeman R. D. (1997). Neural mechanisms underlying binocular fusion and stereopsis: position vs. phase. Proc. Natl. Acad. Sci. U.S.A. 94, 5438–5443. 10.1073/pnas.94.10.5438 9144256 PMC24697

[B3] Chen L. (2021). Adaptive asynchronous control system of robotic arm based on augmented reality-assisted brain-computer interface. J. Neural Eng. 18:066005. 10.1088/1741-2552/ac3044 34654000

[B4] Chen X. Wang Y. Gao S. Jung T.-P. Gao X. (2015). Filter bank canonical correlation analysis for implementing a high-speed SSVEP-based brain-computer interface. J. Neural Eng. 12:046008. 10.1088/1741-2560/12/4/046008 26035476

[B5] Cheng M. Gao X. Gao S. Xu D. (2002). Design and implementation of a brain-computer interface with high transfer rates. IEEE Trans. Biomed. Eng. 49, 1181–1186. 10.1109/TBME.2002.803536 12374343

[B6] Cotrina A. Benevides A. B. Castillo-Garcia J. Benevides A. B. Rojas-Vigo D. Ferreira A.. (2017). A SSVEP-BCI setup based on depth-of-field. IEEE Trans. Neural Syst. Rehabil. Eng. 25, 1047–1057. 10.1109/TNSRE.2017.2673242 28252409

[B7] Duan J. Li S. Ling L. Zhang N. Meng J. (2022). Exploring the effects of head movements and accompanying gaze fixation switch on steady-state visual evoked potential. Front. Hum. Neurosci. 16:943070. 10.3389/fnhum.2022.943070 36171871 PMC9510612

[B8] Guo M. Yue K. Hu H. Lu K. Han Y. Chen S.. (2022). Neural research on depth perception and stereoscopic visual fatigue in virtual reality. Brain Sci. 12:1231. 10.3390/brainsci12091231 36138967 PMC9497221

[B9] Han C. Xu G. Jiang Y. Wang H. Chen X. Zhang K.. (2019). “Stereoscopic motion perception research based on steady-state visual motion evoked potential,” in 2019 41st Annual International Conference of the IEEE Engineering in Medicine and Biology Society (EMBC) (Berlin: IEEE), 3067–3070. 10.1109/EMBC.2019.8857487 31946535

[B10] Hong S. Berkeley B. Kim S. S. (2005). Motion image enhancement of lcds. IEEE Int. Conf. Image Process. 2, II-17. 10.1109/ICIP.2005.1529980

[B11] Jacob R. J. (1991). The use of eye movements in human-computer interaction techniques: what you look at is what you get. ACM Transact. Inf. Syst. 9, 152–169. 10.1145/123078.128728

[B12] Jin J. Xiao R. Daly I. Miao Y. Wang X. Cichocki A. (2020). Internal feature selection method of CSP based on L1-norm and dempster-shafer theory. IEEE Trans. Neural Netw. Learn. Syst. 32, 4814–4825. 10.1109/TNNLS.2020.3015505 32833646

[B13] Kaspar K. Hassler U. Martens U. Trujillo-Barreto N. Gruber T. (2010). Steady-state visually evoked potential correlates of object recognition. Brain Res. 1343, 112–121. 10.1016/j.brainres.2010.04.072 20450897

[B14] Ke Y. Liu P. An X. Song X. Ming D. (2020). An online SSVEP-BCI system in an optical see-through augmented reality environment. J. Neural Eng. 17:016066. 10.1088/1741-2552/ab4dc6 31614342

[B15] Kim H. R. Angelaki D. E. DeAngelis G. C. (2016). The neural basis of depth perception from motion parallax. Philos. Trans. R. Soc. Lond. B Biol. Sci. 371:20150256. 10.1098/rstb.2015.0256 27269599 PMC4901450

[B16] Kurita T. (2001). 35.1: moving picture quality improvement for hold-type am-lcds. SID Symp. Digest Tech. Pap. 32, 986–989. 10.1889/1.1832037

[B17] Li R. Hu H. Zhao X. Wang Z. Xu G. (2023). A static paradigm based on illusion-induced VEP for brain-computer interfaces. J. Neural Eng. 20:026006. 10.1088/1741-2552/acbdc0 36808912

[B18] Liang L. Bin G. Chen X. Wang Y. Gao S. Gao X. (2021). Optimizing a left and right visual field biphasic stimulation paradigm for SSVEP-based BCIs with hairless region behind the ear. J. Neural Eng. 18:066040. 10.1088/1741-2552/ac40a1 34875637

[B19] Lin Z. Zhang C. Wu W. Gao X. (2006). Frequency recognition based on canonical correlation analysis for SSVEP-based BCIs. IEEE Trans. Biomed. Eng. 53, 2610–2614. 10.1109/TBME.2006.886577 17152442

[B20] Liu B. Huang X. Wang Y. Chen X. Gao X. (2020). BETA: a large benchmark database toward SSVEP-BCI application. Front. Neurosci. 14:627. 10.3389/fnins.2020.00627 32655358 PMC7324867

[B21] Lotte F. Bougrain L. Cichocki A. Clerc M. Congedo M. Rakotomamonjy A.. (2018). A review of classification algorithms for eeg-based brain-computer interfaces: a 10 year update. J. Neural Eng. 15:031005. 10.1088/1741-2552/aab2f2 29488902

[B22] Ma X. Yao Z. Wang Y. Pei W. Chen H. (2018). “Combining brain-computer interface and eye tracking for high-speed text entry in virtual reality,” in 23rd International Conference on Intelligent User Interfaces (Tokyo), 263–267. 10.1145/3172944.3172988

[B23] Mahmood M. Kim N. Mahmood M. Kim H. Kim H. Rodeheaver N.. (2022). VR-enabled portable brain-computer interfaces via wireless soft bioelectronics. Biosens. Bioelectron. 210:114333. 10.1016/j.bios.2022.114333 35525171

[B24] Mu J. Grayden D. B. Tan Y. Oetomo D. (2020). “Comparison of steady-state visual evoked potential (SSVEP) with LCD vs. LED stimulation,” in 2020 42nd Annual International Conference of the IEEE Engineering in Medicine & Biology Society (EMBC) (Montreal, QC: IEEE), 2946–2949. 10.1109/EMBC44109.2020.9175838 33018624

[B25] Mun S. Park M.-C. Park S. Whang M. (2012). SSVEP and ERP measurement of cognitive fatigue caused by stereoscopic 3D. Neurosci. Lett. 525, 89–94. 10.1016/j.neulet.2012.07.049 22884933

[B26] Mun S. Park M.-C. Yano S. (2013). Performance comparison of a SSVEP BCI task by individual stereoscopic 3D susceptibility. Int. J. Hum.-Comput. Interact. 29, 789–797. 10.1080/10447318.2013.765289

[B27] Nakanishi M. Wang Y. Chen X. Wang Y.-T. Gao X. Jung T.-P. (2017). Enhancing detection of SSVEPs for a high-speed brain speller using task-related component analysis. IEEE Trans. Biomed. Eng. 65, 104–112. 10.1109/TBME.2017.2694818 28436836 PMC5783827

[B28] Niu L. Bin J. Wang J. K. S. Zhan G. Jia J. Zhang L.. (2023). Effect of 3D paradigm synchronous motion for SSVEP-based hybrid BCI-VR system. Med. Biol. Eng. Comput. 61, 2481–2495. 10.1007/s11517-023-02845-8 37191865

[B29] Qu J. Wang F. Xia Z. Yu T. Xiao J. Yu Z.. (2018). A novel three-dimensional P300 speller based on stereo visual stimuli. IEEE Trans. Hum Mach. Syst. 48, 392–399. 10.1109/THMS.2018.2799525

[B30] Wang Y. Chen X. Gao X. Gao S. (2016). A benchmark dataset for SSVEP-based brain-computer interfaces. IEEE Trans. Neural Syst. Rehabil. Eng. 25, 1746–1752. 10.1109/TNSRE.2016.2627556 27849543

[B31] Wolpaw J. Birbaumer N. Heetderks W. J. McFarland D. J. Peckham P. H. Schalk G.. (2000). Brain-computer interface technology: a review of the first international meeting. IEEE Trans. Neural Syst. Rehabil. Eng. 8, 164–173. 10.1109/TRE.2000.847807 10896178

[B32] Wu Z. Wu Z. (2017). Functional symmetry of the primary visual pathway evidenced by steady-state visual evoked potentials. Brain Res. Bull. 128, 13–21. 10.1016/j.brainresbull.2016.11.005 27845170

[B33] Xiao X. Xu M. Han J. Yin E. Liu S. Zhang X.. (2021). Enhancement for P300-speller classification using multi-window discriminative canonical pattern matching. J. Neural Eng. 18:046079. 10.1088/1741-2552/ac028b 34096888

[B34] Yan W. Xu G. Li M. Xie J. Han C. Zhang S.. (2017). Steady-state motion visual evoked potential (SSMVEP) based on equal luminance colored enhancement. PLoS ONE 12:e0169642. 10.1371/journal.pone.0169642 28060906 PMC5218567

[B35] Yin E. Zhou Z. Jiang J. Yu Y. Hu D. (2015). A dynamically optimized SSVEP brain-computer interface (BCI) speller. IEEE Trans. Biomed. Eng. 62, 1447–1456. 10.1109/TBME.2014.2320948 24801483

[B36] Yu Y. Liu Y. Yin E. Jiang J. Zhou Z. Hu D. (2019). An asynchronous hybrid spelling approach based on EEG-EOG signals for Chinese character input. IEEE Trans. Neural Syst. Rehabil. Eng. 27, 1292–1302. 10.1109/TNSRE.2019.2914916 31071045

[B37] Yue L. Xiao X. Xu M. Chen L. Wang Y. Jung T.-P.. (2020). “A brain-computer interface based on high-frequency steady-state asymmetric visual evoked potentials,” in 2020 42nd Annual International Conference of the IEEE Engineering in Medicine & *Biology Society (EMBC)* (Montreal, QC: IEEE), 3090–3093. 10.1109/EMBC44109.2020.9176855 33018658

[B38] Zehra S. R. Mu J. Syiem B. V. Burkitt A. N. Grayden D. B. (2023). Evaluation of optimal stimuli for ssvep-based augmented reality brain-computer interfaces. IEEE Access 11, 87305–87315. 10.1109/ACCESS.2023.3297882

[B39] Zhang R. Cao L. Xu Z. Zhang Y. Zhang L. Hu Y.. (2023). Improving AR-SSVEP recognition accuracy under high ambient brightness through iterative learning. IEEE Trans. Neural Syst. Rehabil. Eng. 31, 1796–1806. 10.1109/TNSRE.2023.3260842 37030737

[B40] Zhang R. Xu Z. Zhang L. Cao L. Hu Y. Lu B.. (2022). The effect of stimulus number on the recognition accuracy and information transfer rate of SSVEP-BCI in augmented reality. J. Neural Eng. 19:036010. 10.1088/1741-2552/ac6ae5 35477130

[B41] Zhu S. Yang J. Ding P. Wang F. Gong A. Fu Y. (2023). Optimization of ssvep-bci virtual reality stereo stimulation parameters based on knowledge graph. Brain Sci. 13:710. 10.3390/brainsci13050710 37239182 PMC10216479

